# Estrogen and Thyroid Hormone Receptor Activation by Medicinal Plants from Bahia, Brazil

**DOI:** 10.3390/medicines5010008

**Published:** 2018-01-15

**Authors:** Luã Tainã Costa Reis, Magnus Régios Dias da Silva, Silvia Lima Costa, Eudes da Silva Velozo, Ronan Batista, Suzana Telles da Cunha Lima

**Affiliations:** 1 Laboratory of Bioprospection and Biotechnology (LaBBiotec), Institute of Biology, Federal University of Bahia (UFBA), Barão de Jeremoabo Street, 147-Ondina, Salvador, BA 40170-115, Brazil; lua.taina@hotmail.com; 2 Laboratory of Molecular and Translational Endocrinology, Department of Medicine, Federal University of São Paulo (UNIFESP), R. Sena Madureira, 1500-Vila Clementino, São Paulo, SP 04021-001, Brazil; silvamagnus@gmail.com; 3 Laboratory of Neurochemistry and Cell Biology, Department of Biofunction, Institute of Health Sciences, Federal University of Bahia (UFBA), Reitor Miguel Calmon Avenue, 1272-Canela, Salvador, BA 40231-300, Brazil; costasl@ufba.br; 4 Laboratory of Research in Materia Medica, Department of Medicament, Faculty of Pharmacy, Federal University of Bahia (UFBA), Barão de Jeremoabo Street, 147-Ondina, Salvador, BA 40170-115, Brazil; euvelozo@ufba.br; 5 Department of Organic Chemistry, Institute of Chemistry, Federal University of Bahia (UFBA), Barão de Jeremoabo Street, 147-Ondina, Salvador, BA 40170-115, Brazil; ronbatis@ufba.br

**Keywords:** nuclear receptor, estrogen hormone receptor, thyroid hormone receptor, metabolic disorders, Brazilian medicinal plants

## Abstract

**Background**: A number of medicinal plants are traditionally used for metabolic disorders in Bahia state, Brazil. The aim of this study was to evaluate the estrogen receptor (ER) and thyroid receptor (TR) activation of crude extracts prepared from 20 plants. **Methods**: Species were extracted and assayed for receptor activation through both ER and TR gene-reporter assays, using 17β-estradiol and triiodothyronine (T3), respectively, as the positive controls. **Results**: *Cajanus cajan* (Fabaceae), *Abarema cochliacarpus* (Fabaceae), and *Borreria verticillata* (Rubiaceae) were able to activate ER as much as the positive control (17β-estradiol). These three plant species were also assayed for TR activation. At the concentration of 50 µg/mL, *C. cajans* exerted the highest positive modulation on TR, causing an activation of 59.9%, while *B. verticillata* and *A. cochliacarpus* caused 30.8% and 23.3%, respectively. **Conclusions**: Our results contribute towards the validation of the traditional use of *C. cajans*, *B. verticillata*, and *A. cochliacarpus* in the treatment of metabolic disorders related to ER and TR functions. The gene-reporter assay was proven effective in screening crude plant extracts for ER/TR activation, endorsing this methodology as an important tool for future bioprospection studies focused on identifying novel starting molecules for the development of estrogen and thyroid agonists.

## 1. Introduction

Nuclear receptors (NRs) are proteins that form a superfamily of eukaryotic transcription factors; their function is dependent on ligand binding, and they are evolutionarily correlated. The relevance of this group of proteins in metabolic disease is shown by numerous synthetic ligands used in the clinic or under exploratory development for the treatment of diabetes mellitus, dyslipidemia, hypercholesterolemia, or other metabolic abnormalities [1]. These transcriptional activators play an important role in cell signaling through signal transduction in biological responses and on regulatory activity of gene expression over specific targets. This function is performed through the recruitment of co-regulator complexes in genome-specific sites in response to the binding of small molecules such as steroids and thyroid hormones, retinoic acid, vitamin D, fatty acids, and eicosanoids [2,3]. Nuclear receptors display a common molecular structure consisting of an amino terminal region (NTD), with variable amino acid sequence and length, a DNA-binding domain (DBD) which is quite conserved, a ligand-binding domain (LBD) which binds the ligand molecules specific to each receptor, and a region that connects the DBD with LBD, called Hinge (H) [2,4].

The receptor of estrogen hormone (ER) through 17β-estradiol participates in several physiological functions, including the development of secondary sexual characteristics, actions in the vascular system, energetic and bone metabolism, as well as implications in pathologies such as breast cancer that are also strongly associated with control by the receptor [5,6]. On the other hand, thyroid hormone receptors (TRs) are disseminated by various organs in the human body. They are mainly expressed in the liver, pituitary, inner ear, retina, and several brain areas, playing an important role in growth, development and differentiation, as well as in the regulation of key metabolic processes, such as lipogenesis, lipolysis, and thermogenesis [7,8,9]. Mutations in these receptors with hotspots in the LBD binding domain are related to thyroid hormone resistance syndrome [10].

Due to the requirement of interactionwith hormones or other ligands to exercise their function, NRs are good therapeutic targets, since synthetic agonist and antagonist molecules can be drawn, controlling the transcriptional function of the receptor. In addition, a promising approach is the search for molecules from medicinal plants that can act by modulating the activity of these receptors.

Natural products have great importance in the prevention and treatment of diseases. They have been associated with humankind since ancient times, being the only alternatives for the treatment of diseases before the 20th century [11,12]. Researchers stated that even though 250–500 thousand species of flowering plants were known at that year (2007), only 5% were registered in the literature for medicinal uses, which highlights the amount of pharmacological information still not explored, even today [13].

Metabolic and immune systems are essential for life, and this is believed to be the reason why they have been evolutionarily conserved throughout species. Consequently, immune response and metabolic regulation are highly integrated and constitute a central homeostatic mechanism, whose functions depends on each other. Within this scenario, a disruption of normal metabolic processes may occur. Thus, metabolic disorders can be defined as a group of pathophysiological conditions in which there is deficiency in the production, metabolism, storage, or transport of biochemically important compounds, leading to a cluster of chronic imbalance disorders—particularly obesity, type 2 diabetes, and cardiovascular disease. These diseases currently constitute a large burden of morbidity and mortality, impacting global human health and welfare [14,15]. According to the National Institutes of Health (NIH) [16], metabolic disorders are closely related to a few risk factors, including obesity, a high triglyceride and a low high-density lipoprotein (HDL) cholesterol level, high blood pressure, and high fasting blood sugar (leading to diabetes).

In this study, twenty medicinal plants indicated for metabolic diseases were selected from a couple of ethnobotanical surveys conducted in Bahia state, Brazil. All selected plant species were assessed for estrogen hormone receptor (ER) activation through the ER gene-reporter assay, and only those which confirmed significant modulatory activity over ER were also assessed for thyroid hormone receptor (TRβ) activation.

## 2. Materials and Methods

### 2.1. Selection of Plant Species

Starting from surveys conducted in both Pataxó indigenous and Salvador communities, Bahia state, Brazil [17,18], this study selected twenty plants indicated for the treatment of metabolic disorders (Table 1). These plant species were collected, and the corresponding voucher specimens were deposited at the Alexandre Leal Costa Herbarium (ALCB) of the Federal University of Bahia (UFBA), Salvador, Bahia, Brazil, under the code listed in Table 1.

### 2.2. Obtaining Extracts

Initially, the used part of each plant species (Table 1) was separately dried in an oven at 50 °C, and then powdered in a mill to a fine grade. This plant material (1 g) was exposed to ethanol 96% thorough soaking (1:9 *w*/*v*) for 48 h, yielding an ethanol solution which followed centrifugation and separation from pellets. All ethanol extracts were assessed for the estrogen receptor (ER) activation through the corresponding reporter gene-assay, and only the most active ones were subjected to the thyroid receptor (TR) activation assay.

In order to prepare extracts for the TR assay, the selected plant material (powder) was first defatted by maceration with hexane and then soaked with methanol at room temperature to afford—after concentration under reduced pressure—the corresponding methanol extract, which was stored at 4 °C until use.

### 2.3. Transformation and Purification

DH5α *Escherichia coli*-competent cell was transformed separately with two CMV plasmids containing the PGK1 (luciferase) gene and the other, the TRβ gene (kindly given by Paul Webb, Diabetes Center—University of California San Francisco), in the concentrations of 1 µg/µL each. Bacteria and plasmids were incubated for 30 min on ice, subject to thermal shock by 1 min at 42 °C, followed by 2 min on ice. Subsequently 250 µL of S.O.C medium (Invitrogen) were added and the mixture incubated for 1.5 h at 37 °C under agitation of 180 rpms. After centrifugation at 2000 rpm, the bacterial pellet was re-suspended in 50 µL of S.O.C and plated in Petri dishes with LB solid medium containing ampicillin (100 µg/mL) for 12 h. A colony was collected and grown in 500 mL LB, containing ampicillin (100 µg/mL) for 12 h. The liquid culture was centrifuged at 2000 rpm, and the plasmids were purified according to the Qiagen Protocol of *Maxi prep Plasmid Purification Kit*.

### 2.4. Human HEK Cells Cultivation

For human HEK cell cultivation, 292 HEK cells were seeded and grown in Dulbecco’s Modified Eagle Medium (DMEM) (GIBCO) pH 7.2 supplemented with 1.7 g of sodium bicarbonate, 5 mL of penicillin/streptomycin 5000 U/mL (GIBCO), and 10 mM HEPES containing 10% fetal bovine serum (FBS) and filtered in Millipore filter 0.2 µm.

### 2.5. ER Reporter GeneAssay

This procedure was adapted from previously reported methodologies [19,20]. Briefly, ethanol extracts were dissolved in ethanol to produce stock solutions at 100 mg/mL. Each of the plasmids (1 μg) was used to transfect Hela cells by electroporation. Plasmids used were CMVs containing either Full Length ER, luciferase gene (PGK1) or β-Gal (transfection control). After incubation for 24 h, a given ethanolic extract (10 μg) was used per well (2 mL) in a 6 well plate. 17β-estradiol (Sigma-Aldrich, Darmstadt, Germany, 1 μM) was used as the positive control. After overnight incubation, the luminescence reading (in RLU) was made in an illuminometer and the control (β-Gal), in a spectrophotometer, at 460 nm. The reading data were plotted in Graph Pad Prism, and the activation coefficients (luciferase) were calculated by comparison to the positive control. Each luminometer reading reflects activation of the receptor by each ethanol extract when compared to the synthetic estrogen.

### 2.6. TR Reporter Gene Assay

Methanol extracts were dissolved in DMSO to prepare stock solutions at 40 mg/mL. Final concentrations tested in cells were 50 and 100 µg/mL in DMEM—which were demonstrated to be the maximum added to cells (100 µg/mL) without causing cell death superior to the positive control with triiodothyronine (T3)—for all plants.

HEK cells were transfected using calcium phosphate method [21]. Cells were grown in 10 mm plates until confluence of 60%. The medium was aspirated and cells released from the bottom with 1 mL of trypsin for 3 min. After homogenization, 2 mL of DMEM medium was added and the mixture centrifuged for 5 min at 1000 rpm. The pellet was resuspended in 1 mL of medium, distributed in the twelve wells, each containing 1 mL of medium. Cells were grown overnight at 37 °C. One hour prior to transfection, the medium was replaced by DMEM without FBS. Twenty-four micrograms of plasmidial DNA (12 µg of PGK1 and 12 µg TRβ) were added to the 76.25 μL of 2 M CaCl_2_ solution, and the final volume of 625 µL was completed with ultra-pure water. This solution was then added to 625 µL of HEPES 0.05 M (1.6 g NaCl, 0.021 g Na_2_HPO_4_, and 1.3 g HEPES in 100 mL water, pH 7.0) and left for 15 min at room temperature. One hundred microliters (100 µL) of the final solution were added to each well in the 12-well plate. The cells then stayed at 37 °C for two hours. Subsequently, the transfection medium was removed, cells were washed twice with phosphate-buffered saline (PBS), and DMEM medium containing 10% FBS was added. Reporter-gene assay was used for evaluation of gene expression regulated by plant extracts. The positive control was triiodothyronine (T3) in a final concentration of 1 nM (Sigma-Aldrich). Readings of activation used a Dual-Glo^®^ luciferase assay system (E2920, PROMEGA, Madison, WI, USA) according to instructions of the company, and results were obtained according to readings in a luminometer (in RLU).

### 2.7. Statistical Analysis

Statistical analysis was performed in Graphpad Prism 5 (Garph Pad Software, La Jolla, CA, USA), and differences among the data obtained were performed with Wilcoxon test with significance values of *p* < 0.05. Evaluations were non-parametric, as paired sample tests, and three analytical replicates per sample. The graphical expression was performed using mean, standard error, and data values converted into percentage in relation to positive control.

## 3. Results and Discussion

**Figure 1:**
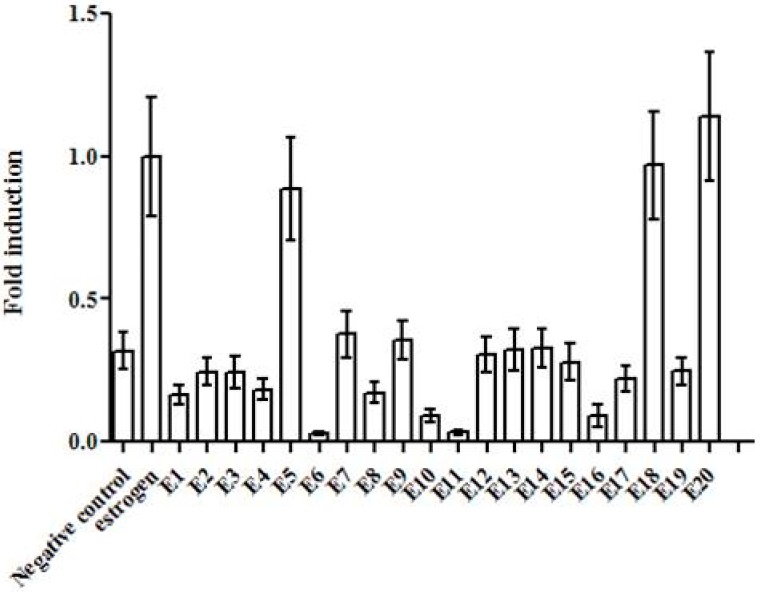
Estrogen receptor (ER) activation by selected ethanol plant extracts. E1: *Cissus verticilata*; E2: *Syzygium cumini*; E3: *Bidens pilosa*; E4: *Petiveria alliaceae*; E5: *Cajanus cajan*; E6: *Rosmarinus officinalis*; E7: *Croton heliotropiifolius*; E8: *Sambucus australis*; E9: *Senna angulata*; E10: *Psidium guajava*; E11: *Maytenus ilicifolia*; E12: *Baccharis trimera*; E13: *Cinchona officinalis*; E14: *Cinnamomum zeylanicum*; E15: *Alpinia nutans*; E16: *Peperomia pellucida*; E17: *Plantago major*; E18: *Abarema cochliacarpus*; E19: *Byrsonima sericea*; E20: *Borreria verticillata*. Data are displayed in average fold induction compared to the positive control (100%) 17β-Estradiol (estrogen). Bars represent the standard deviation of three repetitions (*n* = 3).

Ethanol extracts obtained from all 20 plants listed in Table 1 were assessed for ER activation, and the results are shown in Figure 1. As can be seen, only the crude extracts E5 (*Cajanus cajan*, Fabaceae), E18 (*Abarema cochliacarpus*, Fabaceae), and E20 (*Borreria verticillata*, Rubiaceae) were able to produce an ER activation comparable to that observed for the positive control 17β-estradiol (Figure 1), and for this reason they were classified as active extracts. The ER activation of the remaining ethanol extracts was very similar to or lower than that observed for the negative control, and thus these crude extracts were considered to be inactive.

**Figure 2:**
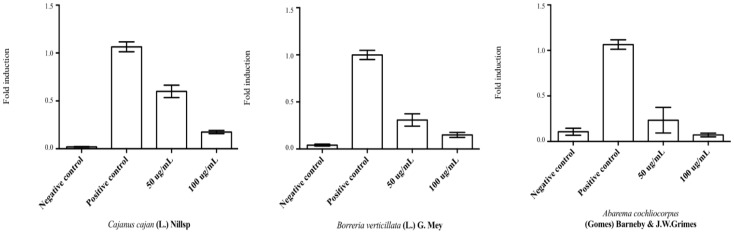
Activation of thyroid receptor β (TRβ) by selected methanol plant extracts. Data are displayed in average fold induction compared to the positive control (100%) triiodothyronine (T3). Bars represent the standard deviation of three repetitions (*n* = 3).

Bearing in mind that estrogen and thyroid receptors crosstalk to each other [22], it was assumed that ER activation might be used as predictor for TR activation. Thus, the three plant species active on ER were selected for the following TR activation assay. Methanol extracts were obtained and assayed for TR activation, and the corresponding results are depicted in Figure 2. Interestingly, all three extracts showed wide variation of TRβ activation, but with increased response at 50 µg/mL compared to 100 µg/mL. These findings reveal that the activation of such receptors is strongly associated with extract concentration. At the concentration of 50 µg/mL, *C. cajans* presented an activation of 59.9%, while *B. verticillata* and *A. cochliacarpus* did 30.8% and 23.3%, respectively. At 100 µg/mL, the corresponding values were respectively 17.4%, 14.9%, and 7.1% (Figure 2). In agreement with these results, the crude ethanol extract of *C. cajans* (L.) Millsp. was demonstrated to exert higher positive modulation on TR when compared to *B. verticillata* and *A. cochliacarpos*.

Plant molecules with modulator roles over receptors are important not only due to the great ability to produce biomolecules with different structures, but also for their contribution to the knowledge of compounds produced by flora [23,24]. Many molecules from natural sources have already been described in the literature as modulators of nuclear receptors [25,26,27]. Genistein, an isoflavone implicated as causative for the estrogenic activity of red clover [28], is one of the known chemical components of *C. cajan* [29] and may explain—at least in part—both ER and TR activation observed for this plant in the present study. *B. verticillata* contains iridoids as chemical constituents [30,31], which are a recognized class of natural products which actively modulate estrogen receptors [32]. Finally, *A. cochliacarpos* is a plant species rich in catechins and polyphenols, which are probably the components of this plant which are active on the ER.

Although there is an increasing interest in isolating active compounds, plant extracts have innumerable molecules that may act synergistically in modulating nuclear receptors, as demonstrated by Lin [33]. In addition to that, extracts have the ability to increase the nuclear receptor activity—even when it is saturated with endogenous ligands—through binding outside the hormone pocket, in allosteric response, as seen by Ong and Tan [25] for androgenic hormone receptor. Products of natural origin—whether from plant, animal, or microbial—represent an almost inexhaustible source of molecular structures that can be useful for therapeutic purposes, or as a model to design new chemical molecules. The results achieved in the present work are of utmost relevance within the field of natural products and nuclear receptors, since it is the first time a plant extract has been reported to exhibit thyroid hormone receptor modulation.

Our findings contribute towards the validation of the traditional use of *Cajanus cajans*, *Borreria verticillata*, and *Abarema cochliacarpus* in the treatment of metabolic disorders—mainly those related to ER and TR functions. Moreover, the gene-reporter assays for ER and TR described in this study were proved effective to screen crude plant extracts for ER/TR activation, and reveal that this methodology may display an important role in future bioprospection studies focused on identifying novel chemical entities as starting molecules for the development of estrogen and thyroid agonists.

## Figures and Tables

**Table 1:** Plants used in Bahia, Brazil, referred to as having therapeutic properties against metabolic disorders.

Code	Species	Family	Voucher n° (ALCB)	Vernacular Name	Indication	Part Used
E1	*Cissus verticilata* L.	Vitaceae	102,060	Insulina	hypertension, diabetes	leaf
E2	*Syzygium cumini* (L.) Skeels	Myrtaceae	76,156	Jamelão	obesity, diabetes	leaf
E3	*Bidens pilosa* L.	Asteraceae	23,464	Picão, Carrapixo	diabetes, menstrual disorders	whole plant
E4	*Petiveria alliaceae* L.	Phytolaccaceae	123,358	Guine	diabetes	leaf
E5	*Cajanus cajan* (L.) Millsp	Fabaceae	76,098	Feijão Guandu	diabetes	leaf, flower
E6	*Rosmarinus officinalis* L.	Lamiaceae	76,128	Alecrim do Reino	metabolic syndrome, human fertility	leaf
E7	*Croton heliotropiifolius* Kunth	Euphorbiaceae	108,456	Caçutinga	diabetes, Alzheimer, Parkinson	flowers and leaf
E8	*Sambucus australis* Cham.	Caprifoliaceae	76,145	Sabugueiro	abdominal adiposity; obesity	stem, flowers
E9	*Senna angulata* (Vogel)	Fabaceae	12,375 *	Sene	obesity	bark, seed
E10	*Psidium guajava* Raddi	Myrtaceae	78,144	Goiabeira	diabetes	leaf, buds
E11	*Maytenus ilicifolia* (Schrad.)	Celastraceae	92,411	Espinheira-Santa	obesity	leaf
E12	*Baccharis trimera* (Less.) DC	Asteraceae	76,132	Carqueja	obesity, diabetes	aerial parts
E13	*Cinchona officinalis* L.	Rubiaceae	12,010	Quina-Quina	cholesterol disorders, obesity	leaf
E14	*Cinnamomum zeylanicum* Blume	Lauraceae	76,099	Canela	cardiopathies, diabetes	bark
E15	*Alpinia nutans* Roscoe	Zingiberaceae	76,123	Levante	metabolic disorders	leaf, rhizome
E16	*Peperomia pellucida* (DC.) H.B.K.	Piperaceae	78,145	Alfavaca-de-cobra	cholesterol disorders, obesity, diabetes	leaf, seed
E17	*Plantago major* (L.)	Plantaginaceae	95,608	Trancagem	diabetes	leaf and seeds
E18	*Abarema cochliacarpus* (Gomes) Barneby & J.W. Grimes	Fabaceae	76,158	Barbatimão	obesity, diabetes	whole plant
E19	*Byrsonima sericea* DC.	Malpighiaceae	78,150	Murici	obesity	leaf
E20	*Borreria verticillata* (L.) G. Mey.	Rubiaceae	76,113	Tiririca de Babado	obesity, diabetes, fever and flu	leaf

* Housed at the Herbarium of the Federal University of Pará (UFP), Brazil. ALCB: Alexandre Leal Costa Herbarium.
